# Assessment of risk perception and risk communication regarding COVID-19 among healthcare providers: An explanatory sequential mixed-method study in Bangladesh

**DOI:** 10.12688/f1000research.27129.1

**Published:** 2020-11-16

**Authors:** Marium Salwa, M Atiqul Haque, Muhmammad Ibrahim Ibne Towhid, Sarmin Sultana, Mohammad Tanvir Islam, Md Maruf Haque Khan, Md Titu Miah, Syed Shariful Islam, Syed Moniruzzaman

**Affiliations:** 1 Department of Public Health and Informatics, Bangabandhu Sheikh Mujib Medical University, Dhaka, 1000, Bangladesh; 2 Center for Language Studies, University of Liberal Arts Bangladesh, Dhaka, 1209, Bangladesh; 3 Department of Internal Medicine, Bangabandhu Sheikh Mujib Medical University, Dhaka, 1000, Bangladesh; 4 Department of Medicine, Mugda Medical College, Dhaka, 1214, Bangladesh; 5 Risk and Environmental Studies, Department of Social and Cultural Sciences, Centre for Societal Risk Research, Karlstad University, Karlstad, 65188, Sweden

**Keywords:** risk perception, risk communication, infection prevention and control practice, healthcare providers, COVID-19, Bangladesh

## Abstract

**Background:** Any public health emergency demands adequate risk communication with the vulnerable population along with their optimized perception about the impending risk to ensure proper risk management and crisis control. Hence, this study will be conducted to explore healthcare providers’ perceptions regarding risks of coronavirus disease 2019 (COVID-19), as well as how they are being communicated to about the risk, and how they practice risk reduction measures.

**Methods:** A two-phased explanatory sequential mixed-method study will be conducted among physicians and nurses from randomly selected tertiary healthcare facilities in Dhaka, the capital of Bangladesh. In the first phase, the general pattern and quantifiable measures of risk perception, risk communication, and infection prevention practices will be assessed quantitatively. Multiple linear regression analyses will be performed to explore how much variability of risk perception is predicted by risk communication methods and contents. In the second phase, qualitative data will be collected for in-depth understanding and exploration of participants’ experiences and insights regarding COVID-19 risk through interviews and document reviews. Thematic content analysis of the qualitative data will be done manually. Findings from both quantitative and qualitative phases will then be triangulated to illustrate the research objectives.

**Discussion:** Based on the psychometric dimensions of risk perception and psycho-social theory of the health belief model, perception of COVID-19 risk among healthcare providers will be evaluated in this study. The relationship between risk perception and infection prevention and control practices among healthcare providers will also be investigated. The explanatory sequential design of this study is expected to generate hypotheses on how risk perception is being shaped in a time of uncertainty and thus, will help to build a proper risk communication strategy to minimize risk perception among healthcare providers.

## Introduction

### Background

The role of health professionals is crucial during an outbreak such as in the current coronavirus disease 2019 (COVID-19) pandemic to maintain population health and provide assurance in retaining healthcare system order. Hence, a clear understanding of how healthcare providers are being communicated with about the risk and how they perceive the risk is essential for emergency preparedness and crisis management ^1^ during public health emergencies.

When risk is the anticipation of a catastrophe, perception applies to the mental processes through which a person deals with the disastrous event ^2^. Studies of risk perception examine the judgments people make when they are asked to characterize and evaluate any hazardous situation ^3^. Empirical studies show that perception and acceptance of risk have their roots embedded in social and cultural factors ^4^ and research evaluating risk perception often offers important pointers concerning the selection of dimensions that need to be focused on for risk management. On the other hand, risk communication is multi-directional communication and engagement with the population at risk, so that they can make informed decisions to protect themselves ^5^. In any health emergency, risk communication is directed to share information essential for saving lives, preserving health, and minimizing harm through changing perception and behaviour ^5^. Communicating risk with healthcare providers is important as it might influence their understanding of the risk, willingness to serve at the frontline and enhance their preventive practices in times of need.

A recent qualitative study in China reported that healthcare providers experienced several challenges while working in COVID-19 wards that include heavy workloads, exhaustion from wearing protective gear, fears of being infected, and a sense of powerlessness while fulfilling their professional responsibilities for patients’ wellbeing ^6^. During the severe acute respiratory syndrome (SARS) epidemic in Japan, a study revealed a high level of risk perception among healthcare providers and hence, emphasized on planning and implementing institutional measures during any health emergency ^7^. Bangladesh reported its first Severe Acute Respiratory Syndrome Coronavirus-2 (SARS-CoV-2) positive case on 8 March 2020, around three months after the first reported case in China. Yet, experiences of Bangladeshi healthcare providers during the COVID-19 pandemic remain mostly unexplored. With a steep rise in new COVID-19 cases in Bangladesh, many healthcare providers have already been infected. Under the circumstances, a clear understanding of different perspectives of disease risk and prevention is needed in order to develop effective prevention strategies ^8^. A detailed understanding of risk perception is also essential for effective risk communication and risk management ^9^. This study has been designed to examine the communication made with healthcare providers and their perceptions regarding the risks related to COVID-19.

### Research questions

1. How do healthcare providers perceive risks related to the ongoing COVID-19 pandemic in Bangladesh?
2. What are the communication channels, influencers, and content used for communicating COVID-19 risk with the healthcare providers in Bangladesh?
3. How do healthcare providers engage in COVID-19 infection prevention and control practices in healthcare settings?
4. How risk perception is being shaped by the nature of risk communication among healthcare providers at the time of the COVID-19 pandemic?

## Methods

### Study design

This will be a two-phased explanatory sequential mixed-method study. According to the study design, a quantitative cross-sectional study will be conducted in the first phase to evaluate the general pattern and quantifiable measures of the research objectives. In the second phase, qualitative data will be collected for in-depth understanding and exploration of participants’ experiences and insights. Data from both quantitative and qualitative phases will then be triangulated to illustrate the answer to each research question.

### Study settings and study population

Registered physicians and nurses working at different tertiary care hospitals in Dhaka, the capital of Bangladesh, will be invited. More than half of the COVID-19 patients in Bangladesh are concentrated in Dhaka ^10^. Here, some healthcare facilities have been dedicated for the treatment of COVID-19 patients while others are open to all patients. Using a lottery method, six facilities will be selected as study sites from the list of public tertiary healthcare facilities in Dhaka.

### Participant recruitment and data collection

***First phase: Quantitative data collection.*** The sample size has been calculated by 4pq/L2. Considering the perception of 50% (p), q as 1− p, 5% allowable error (L), 95% confidence interval, and 10% non-response rate, the calculated sample size is 440 participants. Thus, recruiting at least 440 participants will be adequate for this study. 

During the ongoing COVID-19 pandemic, healthcare providers in tertiary care hospitals in Dhaka are working in shifts following a duty roster. For this purpose, physicians and nurses working for each hospital have been divided into several groups. Usually, one group is working continuously for a pre-fixed period and then going into quarantine as another group replaces them. Considering this context, randomly selected physicians and nurses serving at the selected hospitals or departments of hospitals during the pre-fixed data collection period will be approached.

The following selection criteria will be applied for this study.

*Inclusion criteria*

- Physicians and nurses with valid registration numbers
- Physicians and nurses directly serving patients at any selected hospital within the study period

*Exclusion criteria*

- Physicians and nurses who are not in physical contact with patients during the study period

A list of physicians and nurses will be prepared from the selected hospitals, applying the selection criteria for collecting quantitative data, and a self-administered questionnaire will be distributed among them. A group of data collectors will be trained on the questionnaire beforehand. Data collectors will be made available at the hospitals throughout the data collection period for any clarification regarding the questionnaire.

***Second phase: Qualitative data collection.*** Qualitative data will be collected through in-depth interviews (IDIs) and document review. A strategic sampling strategy with gender balance will be followed for qualitative data collection. Primarily, ten physicians and ten nurses working at the sampled hospitals will be selected through purposive and snowball sampling for interview. Qualitative data will be collected until data saturation.

At first, the selected potential participants will be sent informed consent forms and a permission letter from the corresponding hospital administration asking for their participation in this study. After obtaining their written approval on consent forms, interviews will officially proceed. Secluded places within hospital premises or adjacent to hospitals as per the convenience of the participant will be the preferred interview locations. Maintaining proper physical distancing and other personal protective measures, IDIs will be conducted and digitally recorded. In addition, verbal and non-verbal expressions of the participants will be recorded by note taking. All audio-taped interviews will be transcribed verbatim immediately after each interview.

Documents mentioned by the participants during the interview that need further exploration to accomplish research objectives will also be reviewed.

The data collection plan for collecting qualitative data is shown in Table 1.

**Table 1.** Qualitative data collection plan.

Sl no.	Participant			Type of data collection
	Type	Sex	Number	
1.	Physician	Male	5	IDI Document review
		Female	5	
2.	Nurse	Male	5	IDI Document review
		Female	5	

### Data collection tool

***First phase: Quantitative data collection tool.*** For quantitative data, a structured questionnaire will be constructed encompassing three aspects of risk perception - cognitive, affective, and psychometric. Cognitive risk perception will be assessed by asking the participants to rate their perceived susceptibility to and perceived severity of COVID-19 using a Likert scale. “Standard questionnaire on risk perception of an infectious disease outbreak” developed by the Municipal Public Health Service Rotterdam-Rijnmond ^11^ and constructs of the health belief model (HBM) ^12^ will be followed to set up the questionnaire on cognitive risk perception. The affective dimension of risk perception will be evaluated through fear, anxiety, trust, and general concerns about COVID-19. To evaluate fear, the Fear of COVID-19 Scale ^13^, a well-validated tool will be used. A validated Bengali version of this tool is also available ^14^. Permission has already been obtained for using this tool for this study. For psychometric risk perception, the psychometric paradigm suggested by Slovic *et al.* will be used, which focuses on the qualitative dimensions of the perception on COVID-19 such as perceived voluntariness, catastrophic ability, controllability, severity, personal impact, and novelty ^15^. A German risk perception survey questionnaire ^16^ will also be followed to construct the questionnaire for evaluating psychometric paradigm. Questions to evaluate risk communication will be developed based on literature review and supported by the mental theory of risk communication ^1^. Infection prevention and control (IPC) practices will be assessed based on the IPC guideline provided by WHO ^17^ for the healthcare providers. The questionnaire will be pre-tested prior to the data collection among healthcare workers at a primary healthcare facility in Dhaka.

***Second phase: Qualitative data collection tool.*** A semi-structured guide for IDIs will be prepared, focusing on issues mentioned by the participants in the first phase of the study that need additional explanations. Pilot interviews will be conducted to test the questions in the semi-structured guide and necessary modifications will be made before starting the formal interviews, as recommended by Magnusson and Maracek ^18^.

Documents shared by the respondents during IDIs will be reviewed. Participants will be asked for two types of documents that are relevant to the study objectives and will reflect their experiences on their risk perception, risk communication and preventive practices: public documents such as office notices, training manuals, guidelines, or protocols; and private documents such as personal notes or logs. For example, participants will be asked to share their experiences about the methods of risk communication during IDIs. If they mention any documents while describing their experience, these will be sought and reviewed.

The methodological matrix for the study is presented in Table 2.

**Table 2.** Methodological matrix.

Objective	Activity/ indicator	Methods	Tools/ theories	Participants
1. To understand how physicians and nurses perceive risks related to the ongoing COVID-19 pandemic	Cognitive, affective, and psychometric risk perception	Quantitative	Psychometric paradigm of risk perception, constructs of health belief model, Fear of COVID-19 Scale, and trust questions	Physicians and nurses
	Experience and emotions related to the risk perception	Qualitative	Semi-structured guide and documents	Physicians and nurses
2. To examine the communication mediums, influencers, and content physicians and nurses are being communicated with about the risk of COVID-19	Risk communication channels, content, and influencers that are trusted, preferred, and extensively used	Quantitative	Pre-tested questionnaire	Physicians and nurses
	Experiences related to risk communication	Qualitative	Semi-structured guide and documents	Physicians and nurses
3. To explore the prevention practices of COVID-19 among physicians and nurses	Preventive practices	Quantitative	World Health Organization (WHO) questionnaire	Physicians and nurses
	Experience, challenges, and motivations for prevention practices	Qualitative	Semi-structured guide and documents	Physicians and nurses

### Outcome variables

The outcome variables to be assessed in this study are presented in Table 3.

**Table 3.** Outcome variables to be assessed.

a. Cognitive and affective dimensions of risk perception and constructs of the health belief model	b. Psychometric paradigm of risk perception	c. Risk communication	d. Infection prevention and control (IPC) practices
• Perceived severity of COVID-19 • Perceived susceptibility to COVID-19 and the extent of anxiety • Perceived efficacy of preventive measures, and self-efficacy • Intention to carry out preventive measures • Cues to action • Trust ➢ Trust of hospital administration ➢ Trust of health and public health organizations ➢ Trust of health related government policy makers ➢ Trust of government-provided information on COVID-19 • Fear of COVID-19	• Global recognition of COVID-19 • Whether the risk source can cause a disaster (catastrophic potential) • Ability to personally control the degree of risk • Undesired impact on future generations • Controllability • Certainty of fatal impact should the risk occur (dread) • Increasing risk over time • Perception of being affected personally • Impression on fair distribution of benefit and risk • Voluntary acceptance of the risk • Familiarity with the risk sources • Observable effects • Impression of reversibility of the risk impact • Sensory perception of danger	• Sources of risk information and influencers • Effectiveness and reliability of the sources • Risk communication contents • Clarity, effectivity, practicality, and applicability of information on risk. • Decision making process for serving at hospitals during the pandemic period • Barriers in communicating risk with healthcare providers • Experiences with communication methods • Responses to the crisis	• Use of personal protective equipment and measures • Hand hygiene • Experiences with IPC practices

### Statistical analysis plan

***First phase: Quantitative data analysis.*** Descriptive analysis will be performed for socio-demographic and other professional characteristics. For continuous variables, mean (standard deviation, SD), median, maximum, and minimum will be calculated. Normality assumption will be made by Shapiro-Wilk test and a p-value of less than 5 percent will be considered as an asymmetric distribution. For categorical variables, rate, percentage, and proportion will be calculated. Perceived risk will be assessed using means with SD and comparisons will be made using the student T-test and analysis of variance (ANOVA) test based on different characteristics such as age, sex, profession, living status, type of healthcare facility, etc.

Multiple linear regression analyses will be performed to determine how much variability of risk perception is predicted by mediums, influencers, and content of risk communication, trust, fear, and anxiety. In addition, the association of risk perception and risk prevention practices will be explored through regression analysis. The role of HBM constructs to explain the healthcare providers’ compliance with IPC guidelines will also be analysed through regression analysis. A p < 0.05 will be used as the level of significance. A window-based statistical software package, preferably SPSS-23, will be used for analysis.

***Second phase: Qualitative data analysis.*** Qualitative interviews will be transcribed verbatim immediately after interview and will be checked by two researchers via thorough listening of the interview recordings. Data analysis will start immediately after completion of the first transcript while interviews will still be ongoing. A strategic plan will be developed for analysing interviews and documents, based on the generic coding method proposed by Alase ^19^ and suggestions given by Creswell ^20^. Firstly, a qualitative codebook will be developed based on literature review on the research topics. This codebook will contain a list of potential codes with definitions, examples, and instructions on usage. These codes will provide preliminary guidance on coding process and will be changed based on the information learned in the process of data analysis. Secondly, researchers will read interview transcripts and documents several times, organise responses into block of sentences or statements, condense them into meaningful chunky statements, and list repeatedly expressed words or phrases by the participants. Thus, the codebook will be furnished and applied to all interviews and documents. Thirdly, re-reading of and listening to all the documents and interviews will be done and chunky statements will be condensed into fewer non-repetitive non-overlapping statements and encapsulated to produce the central meaning or meaning units of the interviews and documents. Meaning units will then be grouped into sub-categories and then categories. Consequently, themes will emerge that can answer research questions.

After analysing and evaluating quantitative and qualitative data separately, triangulation or combination of both data types will be done. Data will be compared and converged following Creswell’s guidance ^20^ to increase data validity, reduce potential bias, minimize limitation, and thus, generate in-depth knowledge on research topics.

### Validity/quality assurance strategy

Creswell ^20^ put emphasis on establishing validity of the scores and findings from both quantitative and qualitative measures in any mixed method study. With a view to ensuring the accuracy of the overall study findings, some measures are planned to be executed. A well-calculated and adequate sample size will be deployed in both phases of the study. Findings of the quantitative phase will be carefully analysed to find out potential areas that will need further in-depth explanation and will be included in the qualitative data collection tool. Samples will be drawn from the same population for each phase of the study to validate the outcomes. Two different survey interviewer manuals will be prepared in the local language of Bangladesh for the two phases of data collection. A training session will be organized where an adequate number of data collectors will be trained to introduce themselves, explain the purpose of the study, obtain informed consent, administer the data collection tool, preserve confidentiality, and recognize possible negative reactions and respond properly. Fieldwork activities of data collectors will be monitored and supervised regularly to ensure the validity of data. Every transcript and document will be revised thoroughly by two separate researchers to ensure authenticity and credibility. Codes will be cross-checked by different researchers. Consensus on each meaning unit and study finding will be made by all researchers.

### Ethical statement

Ethical approval for this protocol has been obtained from the Institutional Review Board of Bangabandhu Sheikh Mujib Medical University at its 199th meeting (Memo number- BSMMU/2020/6040). All physical data, transcripts and documents will be coded and stored in locked cabinets to secure participants’ information. Only research personnel will be allowed to access the data. The collected information will be used for research purpose only. Several techniques have been adopted to minimize social, physical, and legal risk during the data collection process. Participants will have the right to withdraw from the research at any time. Each of the participants will be given a special identification number for safeguarding confidentiality and protecting anonymity. An informed consent form will be developed containing detailed information about the aim and objectives of the study, the procedure of the study, benefits and risks of participation and the identity of the principal investigator. Informed written consent to participate in the study will be sought from every respondent in both phases of the study.

### Dissemination

Study findings will be disseminated through an online dissemination seminar. In addition, articles will be written and published in international peer reviewed journals and the data set will be shared in the Mendeley Data repository.

### Study status

Data collection in the first phase of this study has been conducted from 17 to 30 May 2020. The second phase of data collection was completed in August. Now, we are undertaking data analysis and report writing.

## Discussion

Amidst the COVID-19 pandemic, professional requirements have put healthcare professionals into a pressured situation worldwide. Adams & Walls ^21^ describe this situation in two ways- a stressed health system capacity from overwhelming disease burden and vulnerable healthcare providers. In this context, the sequential explanatory design of this mixed-method study will allow assessment of different dimensions of risk perception among healthcare providers in two phases. At first, the distribution and determinants of risk perception will be evaluated in quantifiable measures among study participants. Then, qualitative dimensions of risk perception will be evaluated in-depth through interviews and document reviews. 

In any situation, analysis of the problem and decision making depends on how an individual perceives the risk. People are often found to use heuristic approaches or mental shortcuts for judging and making decisions without much cognitive effort. Slovic & Peters ^22^ showed that in judgement of risk, perception of risk is negatively correlated to the perceived benefit where effects of or feelings for the activity plays a major role. Favourable effects increase the tolerance for that particular risk, especially under pressured circumstances ^23^. Further, negative emotions such as fear and anger are also related to how a risk is perceived by individuals. This study will evaluate the perception of healthcare providers towards the risk of COVID-19 under two major psychological dimensions suggested by Slovic, Fischhoff, & Lichtenstein ^24^: a) dread risk - the extent to which the risk is perceived to have catastrophic potential, feelings of dread and lack of control; and b) unknown risk - the extent to which a risk is judged to be unobservable, unknown, new, or delayed in producing harmful impact.

In this study, cognitive risk perception will be evaluated following the theory of HBM. In cognitive behavioural psychology, human behaviour in response to a risk is influenced by several key constructs such as perceived severity of the risk, perceived susceptibility to the risk, perceived benefits of advised action, perceived barriers in performing advised action, cues to action and self-efficacy, as described in HBM ^12^. HBM theorizes that individuals display healthy behaviour if they accurately perceive the associated risk in terms of both severity and susceptibility ^25^.

This study will use the mental model of risk communication ^1^ to assess how healthcare providers of Bangladesh are being communicated to about the COVID-19 pandemic and how this risk communication affects the perception of risk and resulting preventive behaviors. People, in general, develop a mental model of understanding and interpretation of messages communicated with them based on their cognition ^1^. Furthermore, in any uncertain situation, people generally use heuristics to make decisions, and the utilization of risk information communicated with them greatly depends on the trustworthiness of the information provider ^5, 26^. Thus, when the issue at hand is little known, trust plays a major role in shaping perception and deciding engagement in crisis management and control.

This study will be conducted among physicians and nurses serving at different government hospitals in Dhaka. Thus, the result will not be generalized for healthcare providers working at private hospitals or non-government organizations or hospitals in other parts of the country. A further limitation can be the difference in understanding the questions and Likert scales used in the questionnaire by the participants. To minimise this difference, trained data collectors will be deployed at each study site who can clarify any confusion regarding the questionnaire.

## Conclusion

In the context of the current COVID-19 pandemic, like the rest of the world, Bangladesh is going through a difficult situation where all sectors of the government, especially the health system, are striving to manage the crisis. Thus, evaluating the methods and elements of risk communication, along with different aspects of perceptions of healthcare providers and their preventive practices regarding COVID-19, will help to understand how risk perception is developed during the time of a pandemic crisis.

## Data availability

No data are associated with this article.
