# Assessment of risk perception and risk communication regarding COVID-19 among healthcare providers: An explanatory sequential mixed-method study in Bangladesh

**DOI:** 10.12688/f1000research.27129.2

**Published:** 2022-01-06

**Authors:** Marium Salwa, M Atiqul Haque, Muhmammad Ibrahim Ibne Towhid, Sarmin Sultana, Mohammad Tanvir Islam, Md Maruf Haque Khan, Md Titu Miah, Syed Shariful Islam, Syed Moniruzzaman

**Affiliations:** 1 Department of Public Health and Informatics, Bangabandhu Sheikh Mujib Medical University, Dhaka, 1000, Bangladesh; 2 Center for Language Studies, University of Liberal Arts Bangladesh, Dhaka, 1209, Bangladesh; 3 Department of Internal Medicine, Bangabandhu Sheikh Mujib Medical University, Dhaka, 1000, Bangladesh; 4 Department of Medicine, Mugda Medical College, Dhaka, 1214, Bangladesh; 5 Risk and Environmental Studies, Department of Social and Cultural Sciences, Centre for Societal Risk Research, Karlstad University, Karlstad, 65188, Sweden

**Keywords:** risk perception, risk communication, infection prevention and control practice, healthcare providers, COVID-19, Bangladesh

## Abstract

**Background:** Any public health emergency demands adequate risk communication with the vulnerable population along with their optimized perception about the impending risk to ensure proper risk management and crisis control. Hence, we conducted this study to explore healthcare providers’ perceptions regarding risks of coronavirus disease 2019 (COVID-19), as well as how they are being communicated to about the risk, and how they practice risk reduction measures.

**Methods:** We conducted a two-phased explanatory sequential mixed-method study among physicians and nurses from randomly selected tertiary healthcare facilities in Dhaka, the capital of Bangladesh. In the first phase, we assessed the general pattern and quantifiable measures of risk perception, risk communication, and infection prevention practices quantitatively. We performed multiple linear regression analyses to explore how much variability of risk perception was predicted by risk communication methods and contents. In the second phase, we collected qualitative data for in-depth understanding and exploration of participants’ experiences and insights regarding COVID-19 risk through interviews and document reviews. We manually performed thematic content analysis of the qualitative data. Finally, we triangulated findings from both phases to illustrate the research objectives.

**Discussion:** Based on the psychometric dimensions of risk perception and psycho-social theory of the health belief model, perceptions of COVID-19 risk among healthcare providers were evaluated in this study. The relationship between risk perception and infection prevention and control practices among healthcare providers were also investigated. The explanatory sequential design of this study is expected to generate hypotheses on how risk perception is being shaped in a time of uncertainty and, thus, will help to build a proper risk communication strategy for the healthcare providers

## Introduction

### Background

The role of health professionals is crucial during an outbreak such as in the current coronavirus disease 2019 (COVID-19) pandemic to maintain population health and provide assurance in retaining healthcare system order. Hence, a clear understanding of how healthcare providers are being communicated with about the risk and how they perceive the risk is essential for emergency preparedness and crisis management ^1^ during public health emergencies.

When risk is the anticipation of a catastrophe, perception applies to the mental processes through which a person deals with the disastrous event ^2^. Studies of risk perception examine the judgments people make when they are asked to characterize and evaluate any hazardous situation ^3^. Empirical studies show that perception and acceptance of risk have their roots embedded in social and cultural factors ^4^ and research evaluating risk perception often offers important pointers concerning the selection of dimensions that need to be focused on for risk management. On the other hand, risk communication is multi-directional communication and engagement with the population at risk, so that they can make informed decisions to protect themselves ^5^. In any health emergency, risk communication is directed to share information essential for saving lives, preserving health, and minimizing harm through changing perception and behavior ^5^. Communicating risk with healthcare providers is important as it might influence their understanding of the risk, willingness to serve at the frontline and enhance their preventive practices in times of need.

A recent qualitative study in China reported that healthcare providers experienced several challenges while working in COVID-19 wards that include heavy workloads, exhaustion from wearing protective gear, fears of being infected, and a sense of powerlessness while fulfilling their professional responsibilities for patients’ wellbeing ^6^. During the severe acute respiratory syndrome (SARS) epidemic in Japan, a study revealed a high level of risk perception among healthcare providers and hence, emphasized on planning and implementing institutional measures during any health emergency ^7^. Bangladesh reported its first Severe Acute Respiratory Syndrome Coronavirus-2 (SARS-CoV-2) positive case on 8 March 2020, around three months after the first reported case in China. Yet, experiences of Bangladeshi healthcare providers during the COVID-19 pandemic remain mostly unexplored. With a steep rise in new COVID-19 cases in Bangladesh, many healthcare providers have already been infected. Under the circumstances, a clear understanding of different perspectives of disease risk and prevention is needed to develop effective prevention strategies ^8^. A detailed understanding of risk perception is also essential for effective risk communication and risk management ^9^. This study has been designed to examine the communication made with healthcare providers and their perceptions regarding the risks related to COVID-19.

### Research questions

- How do healthcare providers perceive risks related to the ongoing COVID-19 pandemic in Bangladesh?
- What are the communication channels, influencers, and content used for communicating COVID-19 risk with the healthcare providers in Bangladesh?
- How do healthcare providers engage in COVID-19 infection prevention and control practices in healthcare settings?
- How risk perception is being shaped by the nature of risk communication among healthcare providers at the time of the COVID-19 pandemic?

## Methods

### Study design

This was a two-phased explanatory sequential mixed-method study. According to the study design, a quantitative cross-sectional study was conducted in the first phase to evaluate the general pattern and quantifiable measures of the research objectives. In the second phase, qualitative data were collected for in-depth understanding and exploration of participants’ experiences and insights. Data from both quantitative and qualitative phases were then triangulated to illustrate the answer to each research question.

### Study settings and study population

More than half of the COVID-19 patients in Bangladesh were concentrated in Dhaka during the early phase of COVID-19 pandemic ^10^. Here, some healthcare facilities have been dedicated for the treatment of COVID-19 patients while others are open to all patients. From a list of 8 tertiary level hospitals, 1 university hospital, and 19 specialized hospitals in Dhaka, we selected six hospitals using a lottery method. Among them, one was entirely dedicated for COVID-19 treatment while the rest had dedicated units for COVID-19 patients. We purposively chose three COVID-19 dedicated hospital units and three non-dedicated units as our study setting. We collected data from registered physicians and nurses working at the selected hospital units. 

### Participant recruitment and data collection

***First phase: Quantitative data collection.*** The sample size has been calculated by the formula of 4pq/L2. Considering the perception of 50% (p), q as 1− p, 5% allowable error (L), 95% confidence interval, and 10% non-response rate, the calculated sample size is 440 participants. Thus, recruiting at least 440 participants would be adequate for this study. We expected at least eighty participants from each hospital based on our previous experience, thus we selected six hospitals as our study sites.

The following selection criteria were applied to select potential participant

*Inclusion criteria*

- Physicians and nurses with valid registration numbers
- Physicians and nurses directly serving patients at any selected hospital within the study period

*Exclusion criteria*

- Physicians and nurses who are not in physical contact with patients during the study period, including virologist, pathologists, etc.
- Physicians and nurses in ICU duty

During the ongoing COVID-19 pandemic, physicians and nurses working at tertiary care hospitals have been divided into several groups. Usually, one group is working continuously for a pre-fixed period and then going into quarantine as another group replaces them. We prepared a list of physicians and nurses who were working at the selected hospitals during the data collection period. We approached all healthcare providers on the same duty roster at a time. We distributed self-administered questionnaire along with a consent paper among them. We had a group of trained data collectors who wereavailable at the hospitals throughout the data collection period for any clarification regarding the questionnaire and to collect the completed questionnaire.

***Second phase: Qualitative data collection.*** Qualitative data were collected through in-depth interviews (IDIs) and document review. A strategic sampling strategy with gender balance was followed for qualitative data collection. Primarily, ten physicians and ten nurses working at the sampled hospitals were selected through purposive sampling for interview. Qualitative data were collected until data saturation.

At first, we sent informed consent forms and a permission letter from the corresponding hospital administration to the potential participants asking for their participation in this study. After obtaining their written approval on consent forms, interviews were officially proceed. Secluded places within hospital premises or adjacent to hospitals as per the convenience of the participant were preferred as interview locations. Maintaining proper physical distancing and other personal protective measures, we conducted IDIs and digitally recorded them. In addition, verbal and non-verbal expressions of the participants were recorded by note taking. All audio-taped interviews were transcribed verbatim immediately after each interview.

Documents mentioned by the participants during the interview that needed further exploration to accomplish research objectives were also reviewed.

The data collection plan for collecting qualitative data is shown in Table 1.

**Table 1.** Qualitative data collection plan.

Sl no.	Participant			Type of data collection
	Type	Sex	Number	
1.	Physician	Male	5	IDI Document review
		Female	5	
2.	Nurse	Male	5	IDI Document review
		Female	5	

### Data collection tool

***First phase: Quantitative data collection tool.*** For quantitative data, we constructed a structured questionnaire encompassing three aspects of risk perception - cognitive, affective, and psychometric. Cognitive risk perception was assessed by asking the participants to rate their perceived susceptibility to and perceived severity of COVID-19 using a Likert type scale. “Standard questionnaire on risk perception of an infectious disease outbreak” developed by the Municipal Public Health Service Rotterdam-Rijnmond ^11^ and constructs of the health belief model (HBM) ^12^ were followed to set up the questionnaire on cognitive risk perception. The affective dimension of risk perception was evaluated through fear, anxiety, trust, and general concerns about COVID-19. To evaluate fear, the Fear of COVID-19 Scale ^13^, a well-validated tool was used. A validated Bengali version of this tool is also available ^14^. We obtained permission for using this tool in this study beforehand. For psychometric risk perception, the psychometric paradigm suggested by Slovic *et al.* was used, which focuses on the qualitative dimensions of the perception on COVID-19 such as perceived voluntariness, catastrophic ability, controllability, severity, personal impact, and novelty ^15^. We followed a German risk perception survey questionnaire ^16^ to construct the questionnaire for evaluating psychometric paradigm. We developed questions to evaluate risk communication based on literature review and supported by the mental theory of risk communication ^1^. Infection prevention and control (IPC) practices were assessed based on the IPC guideline provided by WHO ^17^ for the healthcare providers. The questionnaire was pre-tested prior to the data collection among healthcare workers at a primary healthcare facility in Dhaka.

***Second phase: Qualitative data collection tool.*** We prepared a semi-structured guide for IDIs , focusing on issues mentioned by the participants in the first phase of the study that needed additional explanations. Pilot interviews were conducted to test the questions in the semi-structured guide and necessary modifications were made before starting the formal interviews, as recommended by Magnusson and Maracek ^18^.

We also reviewed documents shared by the respondents during IDIs. We asked participants to recommend two types of documents that were relevant to the study objectives and reflected their experiences on their risk perception, risk communication and preventive practices: 1) public documents such as office notices, training manuals, guidelines, or protocols; and 2) private documents such as personal notes or logs. For example, we asked participants to share their experiences about how they were communicated about the risk during the onset of the pandemic. If they mentioned any documents while describing their experience, we sought the document and reviewed it.

The methodological matrix for the study is presented in Table 2.

**Table 2.** Methodological matrix.

Objective	Activity/ indicator	Methods	Tools/ theories	Participants
1. To understand how physicians and nurses perceive risks related to the ongoing COVID-19 pandemic	Cognitive, affective, and psychometric risk perception	Quantitative	Psychometric paradigm of risk perception, constructs of the health belief model, Fear of COVID-19 Scale, and trust questions	Physicians and nurses
	Experience and emotions related to the risk perception	Qualitative	Semi-structured guide and documents	Physicians and nurses
2. To examine the communication mediums, influencers, and content physicians and nurses are being communicated with about the risk of COVID-19	Risk communication channels, content, and influencers that are trusted, preferred, and extensively used	Quantitative	Pre-tested questionnaire	Physicians and nurses
	Experiences related to risk communication	Qualitative	Semi-structured guide and documents	Physicians and nurses
3. To explore the prevention practices of COVID-19 among physicians and nurses	Preventive practices	Quantitative	World Health Organization (WHO) questionnaire	Physicians and nurses
	Experience, challenges, and motivations for prevention practices	Qualitative	Semi-structured guide and documents	Physicians and nurses

### Outcome variables

The outcome variables to be assessed in this study are presented in Table 3.

**Table 3.** Outcome variables to be assessed.

a. Cognitive and affective dimensions of risk perception and constructs of the health belief model	b. Psychometric paradigm of risk perception	c. Risk communication	d. Infection prevention and control (IPC) practices
• Perceived severity of COVID-19 • Perceived susceptibility to COVID-19 and the extent of anxiety • Perceived efficacy of preventive measures, and self-efficacy • Intention to carry out preventive measures • Cues to action • Trust ➢ Trust of hospital administration ➢ Trust of health and public health organizations ➢ Trust of health related government policy makers ➢ Trust of government-provided information on COVID-19 • Fear of COVID-19	• Global recognition of COVID-19 • Whether the risk source can cause a disaster (catastrophic potential) • Ability to personally control the degree of risk • Undesired impact on future generations • Controllability • Certainty of fatal impact should the risk occur (dread) • Increasing risk over time • Perception of being affected personally • Impression on fair distribution of benefit and risk • Voluntary acceptance of the risk • Familiarity with the risk sources • Observable effects • Impression of reversibility of the risk impact • Sensory perception of danger	• Sources of risk information and influencers • Effectiveness and reliability of the sources • Risk communication contents • Clarity, effectivity, practicality, and applicability of information on risk. • Decision making process for serving at hospitals during the pandemic period • Barriers in communicating risk with healthcare providers • Experiences with communication methods • Responses to the crisis	• Use of personal protective equipment and measures • Hand hygiene • Experiences with IPC practices

### Statistical analysis plan

***First phase: Quantitative data analysis.*** Descriptive analysis were performed for socio-demographic and other professional characteristics. For continuous variables, mean (standard deviation, SD), median, maximum, and minimum were calculated. Normality assumption was made by Shapiro-Wilk test and a p-value of less than 5 percent was considered as an asymmetric distribution. For categorical variables, rate, percentage, and proportion were calculated. Perceived risk was assessed using means with SD and comparisons were made using the student T-test and analysis of variance (ANOVA) test based on different characteristics such as age, sex, profession, living status, type of healthcare facility, etc.

Multiple linear regression analyses were performed to determine how much variability of risk perception was predicted by mediums, influencers, and content of risk communication, trust, fear, and anxiety. In addition, the association of risk perception and risk prevention practices was explored through regression analysis. The role of HBM constructs to explain the healthcare providers’ compliance with IPC guidelines was also analyzed through regression analysis. A p < 0.05 was used as the level of significance. A window-based statistical software package, preferably SPSS-23, was used for analysis.

***Second phase: Qualitative data analysis.*** Qualitative interviews were transcribed verbatim immediately after interview and were checked by two researchers via thorough listening of the interview recordings. Data analysis were started immediately after completion of the first transcript while interviews were still going on. A strategic plan was developed for analyzing interviews and documents, based on the generic coding method proposed by Alase ^19^ and suggestions given by Creswell ^20^. Firstly, a qualitative codebook was developed based on literature review on the research topics. This codebook contained a list of potential codes with definitions, examples, and instructions on usage. These codes provided preliminary guidance on coding process and were changed based on the information learned in the process of data analysis. Secondly, researchers read interview transcripts and documents several times, organized responses into block of sentences or statements, condensed them into meaningful chunky statements, and listed repeatedly expressed words or phrases by the participants. Thus, the codebook was furnished and applied to all interviews and documents. Thirdly, re-reading of and listening to all the documents and interviews were done and chunky statements were condensed into fewer non-repetitive non-overlapping statements and encapsulated to produce the central meaning or meaning units of the interviews and documents. Meaning units were then grouped into sub-categories and then categories. Consequently, themes were emerged that can answer research questions.

After analyzing and evaluating quantitative and qualitative data separately, triangulation or combination of both data types was done. Data were compared and converged following Creswell’s guidance ^20^ to increase data validity, reduce potential bias, minimize limitation, and thus, generate in-depth knowledge on research topics.

### Validity/quality assurance strategy

Creswell ^20^ put emphasis on establishing validity of the scores and findings from both quantitative and qualitative measures in any mixed method study. With a view to ensuring the accuracy of the overall study findings, some measures were planned to be executed. A well-calculated and adequate sample size was deployed in both phases of the study. Findings of the quantitative phase were carefully analyzed to find out potential areas that needed further in-depth explanation and were included in the qualitative data collection tool. Samples were drawn from the same population for each phase of the study to validate the outcomes. Two different survey interviewer manuals were prepared in the local language of Bangladesh for the two phases of data collection. A training session was organized where an adequate number of data collectors were trained to introduce themselves, explain the purpose of the study, obtain informed consent, administer the data collection tool, preserve confidentiality, and recognize possible negative reactions and respond properly. Fieldwork activities of data collectors were monitored and supervised regularly to ensure the validity of data. Every transcript and document was revised thoroughly by two separate researchers to ensure authenticity and credibility. Codes were cross-checked by different researchers. Consensus on each meaning unit and study finding was made by all researchers.

### Ethical statement

Ethical approval for this protocol was obtained from the Institutional Review Board of Bangabandhu Sheikh Mujib Medical University at its 199th meeting (Memo number- BSMMU/2020/6040). All physical data, transcripts, and documents were coded and stored in locked cabinets to secure participants’ information. Only research personnel are allowed to access the data. The collected information are used for research purpose only. Several techniques have been adopted to minimize social, physical, and legal risk during the data collection process. Participants had the right to withdraw from the research at any time. Each of the participants was given a special identification number for safeguarding confidentiality and protecting anonymity. An informed consent form was developed containing detailed information about the aim and objectives of the study, the procedure of the study, benefits and risks of participation and the identity of the principal investigator. Informed written consent to participate in the study was sought from every respondent in both phases of the study.

### Dissemination

Study findings will be disseminated through an online dissemination seminar. In addition, articles will be written and published in international peer reviewed journals and the data set will be shared in the Mendeley Data repository.

### Study status

Data collection in the first phase of this study has been conducted from 17 to 30 May 2020. The second phase of data collection was completed in August. Now, we are undertaking data analysis and report writing.

## Discussion

Amidst the COVID-19 pandemic, professional requirements have put healthcare professionals into a pressured situation worldwide. Adams & Walls ^21^ described this situation in two ways- a stressed health system capacity from overwhelming disease burden and vulnerable healthcare providers. In this context, the sequential explanatory design of this mixed-method study allowed assessment of different dimensions of risk perception among healthcare providers in two phases. At first, the distribution and determinants of risk perception were evaluated in quantifiable measures among study participants. Then, qualitative dimensions of risk perception were evaluated in-depth through interviews and document reviews.

In any situation, analysis of the problem and decision making depends on how an individual perceives the risk. People are often found to use heuristic approaches or mental shortcuts for judging and making decisions without much cognitive effort. Slovic & Peters ^22^ showed that in judgement of risk, perception of risk is negatively correlated to the perceived benefit where effects of or feelings for the activity plays a major role. Favorable effects increase the tolerance for that particular risk, especially under pressured circumstances ^23^. Further, negative emotions such as fear and anger are also related to how a risk is perceived by individuals. This study evaluated the perception of healthcare providers towards the risk of COVID-19 under two major psychological dimensions suggested by Slovic, Fischhoff, & Lichtenstein ^15^: a) dread risk - the extent to which the risk is perceived to have catastrophic potential, feelings of dread and lack of control; and b) unknown risk - the extent to which a risk is judged to be unobservable, unknown, new, or delayed in producing harmful impact.

In this study, cognitive risk perception was evaluated following the theory of HBM. In cognitive behavioral psychology, human behavior in response to a risk is influenced by several key constructs such as perceived severity of the risk, perceived susceptibility to the risk, perceived benefits of advised action, perceived barriers in performing advised action, cues to action and self-efficacy, as described in HBM ^12^. HBM theorizes that individuals display healthy behavior if they accurately perceive the associated risk in terms of both severity and susceptibility ^24^.

This study used the mental model of risk communication ^1^ to assess how healthcare providers of Bangladesh are being communicated to about the COVID-19 pandemic and how this risk communication affects the perception of risk and resulting preventive behaviors. People, in general, develop a mental model of understanding and interpretation of messages communicated with them based on their cognition ^1^. Furthermore, in any uncertain situation, people generally use heuristics to make decisions, and the utilization of risk information communicated with them greatly depends on the trustworthiness of the information provider ^5, 25^. Thus, when the issue at hand is little known, trust plays a major role in shaping perception and deciding engagement in crisis management and control.

This study was conducted among physicians and nurses serving at different government hospitals in Dhaka. Thus, the result will not be generalized for healthcare providers working at private hospitals or non-government organizations or hospitals in other parts of the country. A further limitation can be the difference in understanding the questions used in the questionnaire by the participants- physicians and nurses. To minimize this difference, trained data collectors were deployed at each study site who clarified any confusion regarding the questionnaire.

## Conclusion

In the context of the current COVID-19 pandemic, like the rest of the world, Bangladesh is going through a difficult situation where all sectors of the government, especially the health system, are striving to manage the crisis. Thus, evaluating the methods and elements of risk communication, along with different aspects of risk perceptions of healthcare providers and their preventive practices regarding COVID-19, will help to understand how risk perception is developed during the time of a pandemic crisis.

## Data availability

No data are associated with this article.
