# Supplementary material for: Assessment of risk perception and risk communication regarding COVID-19 among healthcare providers: An explanatory sequential mixed-method study in Bangladesh
Source: F1000Res. 2022 Jan 6;9:1335. Originally published 2020 Nov 16. [Version 2] doi: 10.12688/f1000research.27129.2 (PMC8817067; doi:10.12688/f1000research.27129.2)
Supplement: Peer review history

# Peer review: Reviewer response for version 2

**DOI:** 10.5256/f1000research.80116.r118842

**Published:** 2022-02-04

**Authors:** Qaisar Khalid Mahmood

**Affiliations:** 1 Department of Sociology, International Islamic University Islamabad, Islamabad, Pakistan

The authors have incorporated the changes. The paper may be accepted.

Is the study design appropriate for the research question?

Partly

Is the rationale for, and objectives of, the study clearly described?

Yes

Are sufficient details of the methods provided to allow replication by others?

Yes

Are the datasets clearly presented in a useable and accessible format?

Partly

Reviewer Expertise:

Medical Sociology and Public Health

I confirm that I have read this submission and believe that I have an appropriate level of expertise to confirm that it is of an acceptable scientific standard.

# Peer review: Reviewer response for version 2

**DOI:** 10.5256/f1000research.80116.r118843

**Published:** 2022-01-19

**Authors:** Salim Mahmud Chowdhury

**Affiliations:** 1 Centre for Injury Prevention and Research, Dhaka, Bangladesh

I don't have any additional comments and approve this version for indexing.

Is the study design appropriate for the research question?

Yes

Is the rationale for, and objectives of, the study clearly described?

Yes

Are sufficient details of the methods provided to allow replication by others?

Yes

Are the datasets clearly presented in a useable and accessible format?

Yes

Reviewer Expertise:

NA

I confirm that I have read this submission and believe that I have an appropriate level of expertise to confirm that it is of an acceptable scientific standard.

# Peer review: Reviewer response for version 1

**DOI:** 10.5256/f1000research.29969.r82187

**Published:** 2021-07-26

**Authors:** Qaisar Khalid Mahmood

**Affiliations:** 1 Department of Sociology, International Islamic University Islamabad, Islamabad, Pakistan

The study protocol is well designed but there are some concerns need to addressed. 

1. How many healthcare facilities have been dedicated for the treatment of COVID-19 patients.
2. Why are you selecting six facilities?
3. How the sample will be allocated to the selected facilities.
4. Will the researchers consider the professional cadres while collecting the data for physicians and nursers. 

Is the study design appropriate for the research question?

Partly

Is the rationale for, and objectives of, the study clearly described?

Yes

Are sufficient details of the methods provided to allow replication by others?

Yes

Are the datasets clearly presented in a useable and accessible format?

Partly

Reviewer Expertise:

Medical Sociology and Public Health

I confirm that I have read this submission and believe that I have an appropriate level of expertise to confirm that it is of an acceptable scientific standard, however I have significant reservations, as outlined above.

# Response

**Published:** 2021-12-04

**Authors:** Marium Salwa

**Affiliations:** Bangabandhu Sheikh Mujib Medical University, Bangladesh

- Acknowledged and incorporated (study settings and study population).
- The reason for selecting six facilities has been integrated (Participant recruitment and data collection). 
- The sample allocation to the facilities has been described (Participant recruitment and data collection).
- We did not consider physicians' and nurses' professional cadre.

# Peer review: Reviewer response for version 1

**DOI:** 10.5256/f1000research.29969.r87598

**Published:** 2021-07-07

**Authors:** Salim Mahmud Chowdhury

**Affiliations:** 1 Centre for Injury Prevention and Research, Dhaka, Bangladesh

The study design is quite appropriate to answer the research questions. Data analysis plan is well described. However, further explanation of the following issues would be helpful for better understanding:

- Six facilities would be selected for the study, but it is not mentioned whether these hospitals would be dedicated facilities for COVID-19 patient management or not. If it is not dedicated facilities, then inclusion and exclusion criteria need to be specified (it would be COVID-19 patients not all patients);
- Further clarification in selecting qualitative interview participants by snowball sampling approach is needed; and
- Study status shows that data collection is completed. However, it is mentioned in other sections that data will be collected which is confusing.

Is the study design appropriate for the research question?

Yes

Is the rationale for, and objectives of, the study clearly described?

Yes

Are sufficient details of the methods provided to allow replication by others?

Yes

Are the datasets clearly presented in a useable and accessible format?

Yes

Reviewer Expertise:

Public health especially heath system research

I confirm that I have read this submission and believe that I have an appropriate level of expertise to confirm that it is of an acceptable scientific standard, however I have significant reservations, as outlined above.

# Response

**Published:** 2021-12-04

**Authors:** Marium Salwa

**Affiliations:** Bangabandhu Sheikh Mujib Medical University, Bangladesh

- We collected data from both COVID-19 dedicated and non-dedicated hospitals which has been mentioned in the revised manuscript.
- We initially planned a snowball sampling which was not required later. So, we edited the text accordingly.
- The entire manuscript has been revised and edited. 
